# Type I interferons and TGF-**β** cooperate to induce liver fibrosis during HIV-1 infection under antiretroviral therapy

**DOI:** 10.1172/jci.insight.152738

**Published:** 2022-07-08

**Authors:** James Ahodantin, Kouki Nio, Masaya Funaki, Xuguang Zhai, Eleanor Wilson, Shyamasundaran Kottilil, Liang Cheng, Guangming Li, Lishan Su

**Affiliations:** 1 Division of Virology, Pathogenesis, and Cancer, Institute of Human Virology, Departments of Pharmacology and Microbiology and Immunology, University of Maryland School of Medicine, Baltimore, Maryland, USA.; 2 Lineberger Comprehensive Cancer Center, Department of Microbiology and Immunology, The University of North Carolina at Chapel Hill, Chapel Hill, North Carolina, USA.; 3 Division of Clinical Care and Research, Institute of Human Virology, Department of Medicine, University of Maryland School of Medicine, Baltimore, Maryland, USA.

**Keywords:** AIDS/HIV, Inflammation, Fibrosis, Macrophages, Mouse models

## Abstract

Liver diseases have become a major comorbidity health concern for people living with HIV-1 (PLWH) treated with combination antiretroviral therapy (cART). To investigate if HIV-1 infection and cART interact to lead to liver diseases, humanized mice reconstituted with progenitor cells from human fetal livers were infected with HIV-1 and treated with cART. We report here that chronic HIV-1 infection with cART induced hepatitis and liver fibrosis in humanized mice, associated with accumulation of M2-like macrophages (M2LMs), elevated TGF-β, and IFN signaling in the liver. Interestingly, IFN-I and TGF-β cooperatively activated human hepatic stellate cells (HepSCs) in vitro. Mechanistically, IFN-I enhanced TGF-β–induced SMAD2/3 activation in HepSCs. Finally, blockade of IFN-I signaling reversed HIV/cART-induced liver diseases in humanized mice. Consistent with the findings in humanized mice with HIV-1 and cART, we detected elevated markers of liver injury, M2LMs, and of IFN signaling in blood specimens from PLWH compared with those of healthy individuals. These findings identify the IFN-I/M2LM/HepSC axis in HIV/cART-induced liver diseases and suggest that inhibiting IFN-I signaling or M2LM may provide a novel therapeutic strategy for treating HIV/cART-associated liver diseases in PLWH treated with antiretroviral therapy.

## Graphical abstract

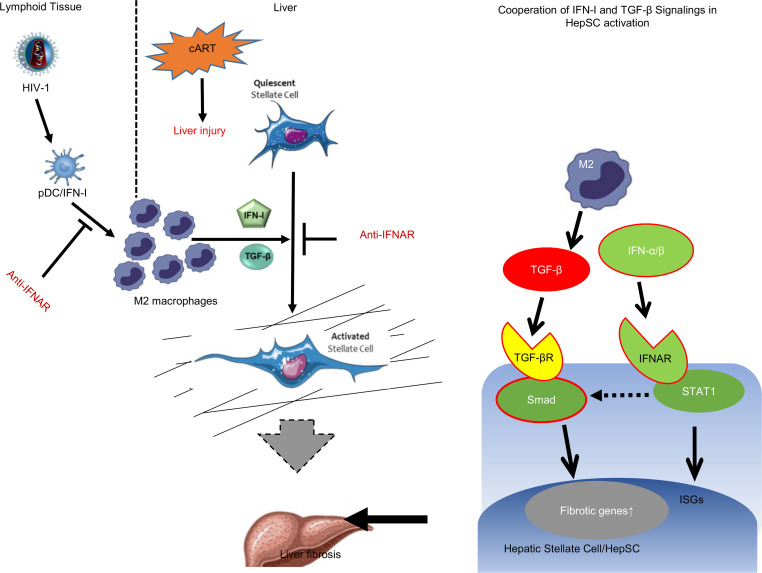

## Introduction

Combination antiretroviral therapy (cART) effectively suppresses HIV-1 replication and significantly improves immune function in HIV-positive individuals and thus has reduced the incidence of AIDS-related mortality and morbidity, especially those associated with profound immunodeficiency (e.g., opportunistic infections) (1). However, these patients with controlled HIV-1 replication are at increased risk for developing several other diseases, among which liver diseases such as liver fibrosis or hepatocellular carcinoma are a leading cause of comortality and comorbidity (2–4). More than 20 million HIV-infected people are currently treated with cART, but liver diseases associated with HIV-1 infection under cART are understudied, due to the lack of a robust animal model. HIV-induced inflammation is not completely resolved during cART and may contribute to the increased risk of liver diseases. Coinfection with hepatitis virus C (HCV) or hepatitis virus B (HBV) contributes to the elevated liver problems in some patients with cART-treated HIV-1 (5–7). In several epidemiological studies of HIV-1 infection, only 50% of HIV-associated liver diseases were attributed to HBV and HCV coinfection or alcohol abuse (8–11). Two other cofactors in HIV-enhanced liver diseases are cART-induced liver injuries and lipotoxic metabolites induced by some of the cART drugs and by nonalcoholic fatty liver diseases (12–15). Indeed, HIV and antiretroviral therapy (ART) induce a loss of mitochondrial function leading to intracellular lipid accumulation, necrosis, and hepatotoxicity (16, 17), and hepatic dysfunction with chronic transaminase elevation, which may contribute to liver diseases in PLWH (18–20).

Type I IFNs (IFN-I) play a critical role in suppressing HIV-1 infection and setting the antiviral host immune program. However, strong correlations have been established between persistently activated IFN signaling with HIV-1 (21) or SIV disease progression (22, 23). During chronic infection, persistent IFN-I signaling is associated with HIV-associated hyperinflammation, immune exhaustion, and HIV-1 persistence. Although IFN-I levels are reduced under cART (24), low levels of IFN-I signaling persist and IFN-stimulated genes (ISGs) are still upregulated in peripheral blood cells or lymphoid organs (25, 26), which may contribute to increased clinical complications and mortality in patients receiving cART for HIV-1 (2). We and others have shown that persistent activation of plasmacytoid DCs (pDCs) (27) and IFN-I signaling during chronic HIV infection in humanized mice (hu-mice) contributes to HIV-induced diseases in all lymphoid organs, even when HIV replication is effectively suppressed by cART (28–31). Intriguingly, blocking IFN-α/β receptor (IFNAR) in cART-treated hu-mice rescued anti-HIV immunity and reduced HIV-1 reservoirs (28, 32).

Macrophages are the key players in various liver diseases, including liver fibrosis and hepatocellular carcinoma. We recently reported (33, 34) that HBV or HCV persistent infection induces liver fibrosis associated with human M2-like macrophages and activation of human hepatic stellate cells (HepSCs) in humanized mouse models. Similar pathogenic cells are detected in human liver samples from patients with HBV or HCV infection (33–35). In patients with HIV-1 infection who are receiving ART, liver macrophages have been reported to be also susceptible to HIV-1 infection and to contribute to persistence of HIV-1 (36–39). We have also reported that depletion of Tregs in HIV-infected hu-mice leads to elevated alanine transaminase (ALT) levels, human macrophage infiltration, and liver fibrosis in hu-mice reconstituted with progenitor cells from human fetal livers (40).

We report here that the combination of HIV-1 infection with cART in hu-mice led to liver diseases with immune cell infiltration and liver fibrosis. These livers were characterized by an accumulation of human M2-like macrophages, induction of IFN-I signaling, activation of HepSCs, and expression of collagens and inhibitors of collagen-degrading enzymes. Furthermore, we discovered that IFN-I directly enhanced TGF-β–induced activation of HepSCs through elevated Smad2/3 phosphorylation in human HepSCs. Finally, blockade of IFN-I signaling in HIV/cART treated mice prevented hepatic immune infiltration and reversed HIV/cART-induced liver diseases.

## Results

### HIV-1 infection combined with cART induces hepatitis and liver fibrosis in hu-mice.

PLWH treated with cART are at increased risk for developing liver diseases such as liver fibrosis. To investigate liver diseases related to HIV-1 infection and cART together, humanized NRG mice reconstituted with CD34^+^ progenitor cells isolated from human fetal livers (NRG-hu HSC mice) were inoculated with HIV-1. From 4 weeks to 12–13 weeks postinfection (wpi), mock- or HIV-1–infected mice were treated with cART, as we have reported (28, 30, 31, 41). The ART doses used in this study were the required doses for effective HIV suppression to undetectable limit in blood and lymphoid tissue, did not induce a spontaneous mouse death for more than 3 months treatment in our mouse model (28, 30, 31, 41) (other data not shown), and were similar to those reported by other investigators (42–44) (Supplemental Table 1; supplemental material available online with this article; https://doi.org/10.1172/jci.insight.152738DS1). Tissues were harvested at 12–13 wpi and analyzed for liver diseases (28, 30, 31, 41) (Supplemental Tables 2–5).

**Figure 1:**
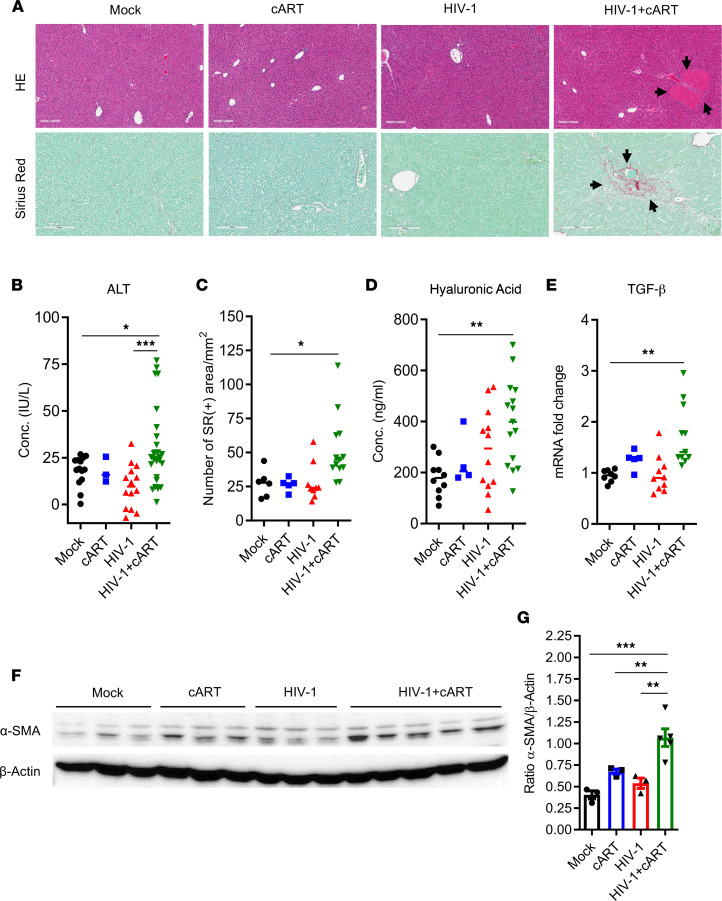
HIV-1 and cART cooperatively induce liver fibrosis in hu-mice. NRG-hu HSC mice were inoculated with PBS or HIV-1 and received cART treatment from 4 through 12 or 13 wpi. Liver tissues from mice were analyzed at 12–13 wpi. (**A**) H&E and SR staining (the latter for fibrosis) of liver sections. Scale bars: 200 μm (top); 100 μm (bottom). (**B**) ALT assessment by ELISA in blood. (**C**) SR/fibrosis quantification by deconvolution algorithm analysis and data are expressed as the number of SR-positive areas per square millimeter. (**D**) Hyaluronic acid detection in the serum. (**E**) Human TGF-β detection in the liver by RT-qPCR and normalized with mouse Gapdh. (**F** and **G**) Analysis of α-SMA expression by (**F**) immunoblot and (**G**) quantification, in the livers of hu-mice infected with HIV-1 and treated with cART and their littermate mock controls. Histograms represent SEM and bars in the scatter plots represent the median value. Statistical analysis was performed with 1-way ANOVA and Turkey’s or Fisher’s LSD post hoc test. *P < 0.05; **P < 0.005; ***P < 0.0005. Conc., concentration.

**Figure 2:**
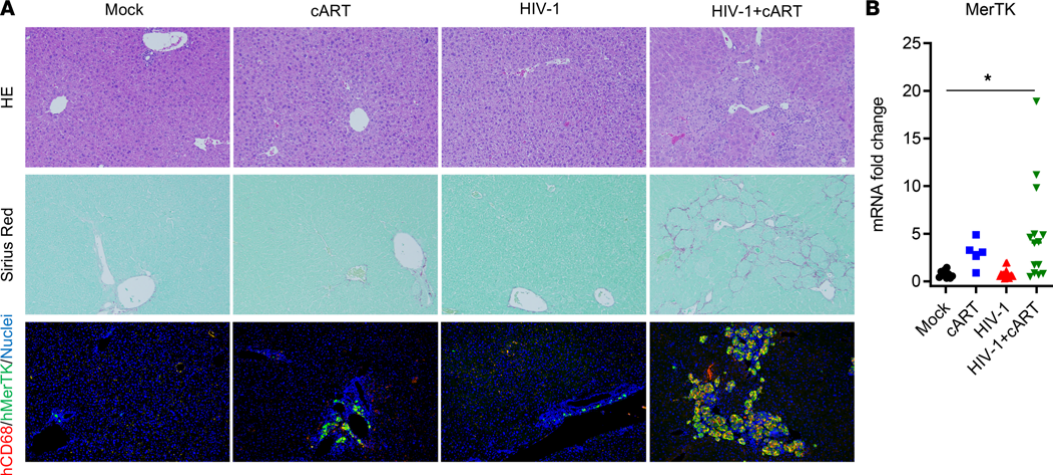
HIV-1 and cART induce M2-like macrophages in the liver of hu-mice. Assessment of human immune cells infiltrated in livers of treated NRG-hu mice collected at 12–13 wpi. (**A**) H&E, SR, and immunofluorescence staining for human CD68 (red), MerTK (green), and nuclei (blue) in the livers of hu-mice (original magnification, ×20). (**B**) RT-qPCR analysis of human MerTK mRNA in the liver. Data were normalized with mouse Gapdh. Bars in the scatter plots represent the median value. Statistical analysis was performed with 1-way ANOVA and Turkey’s post hoc test. *P < 0.05.

Compared with the control mice, H&E staining showed the presence of large necro-inflammatory clusters mainly in the liver of HIV-infected mice treated with cART (HIV/cART) mice (Figure 1A and Figure 2A, top panels), indicating that the combination of HIV-1 infection and cART led to liver inflammation or hepatitis in NRG-hu mice. Consistently, a significant increase in ALT in the serum of HIV-1/cART mice was detected compared with control animals (Figure 1B and Supplemental Figure 8A).

To further characterize the liver pathology induced by HIV/cART, Sirius red (SR) staining was performed on liver sections (Figure 1A and Figure 2A, bottom and middle panels, respectively). We also detected a higher level of collagen deposition in the liver of HIV/cART mice compared with the control mice (Figure 1, A and C). In addition, SR analysis of liver sections revealed 20% of cART-treated mice and 66.7% of HIV/cART mice had collagen deposition in the liver (Supplemental Figure 1A). Compared with the control mice, only HIV/cART mice had a significant increase in hyaluronic acid level in blood (Figure 1D and Supplemental Figure 8B) and TGF-β expression in the liver (Figure 1E and Supplemental Figure 8D), both surrogate markers of liver injury and fibrosis. These observations were strongly supported by the immunoblot analysis of α-SMA proteins showing a higher increase of α-SMA expression in the liver of HIV/cART mice compared with control groups (Figure 1, F and G, and Supplemental Figure 8C). Consistently, we detected expression of fibrosis genes in livers. Compared with mock-infected animals, only HIV/cART mice had a significant increase in mRNA levels of Col.7a1, Timp1, and MMP-13 in the liver (Supplemental Figure 1B). Altogether, our results show that HIV/cART induced liver fibrosis in hu-mice.

### HIV-1 and cART induce hepatic accumulation of M2-like macrophages and IFN-I signaling in the liver.

To further evaluate the liver immunopathogenesis associated with HIV/cART in hu-mice, liver tissues were stained for H&E and for SR and by immunofluorescence for human macrophage infiltration. Compared with control groups, the HIV/cART mice had liver damage associated with high collagen deposition and a massive infiltration of human macrophages (Figure 2A). These hepatic human immune cells in HIV/cART mice were characterized by expression of human CD68 and MerTK, markers of macrophages with M2 phenotype (Figure 2A and Supplemental Figure 2). Consistently, HIV/cART animals also showed a significant increase in expression of MerTK mRNA in the liver (Figure 2B).

**Figure 3:**
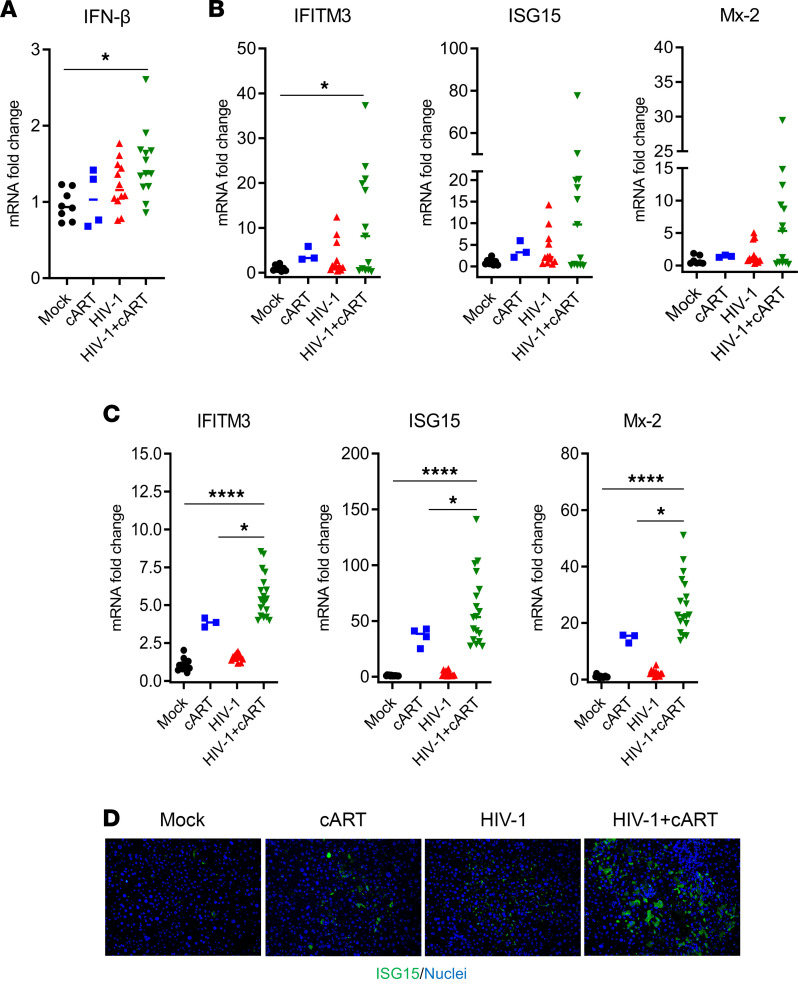
HIV-1 and cART induce IFN-I signaling pathways in the liver of hu-mice. Expression of ISGs in the livers of NRG-hu mice collected at 12–13 wpi. (**A**–**C**) RT-qPCR analysis in the liver of (**A** and **B**) human genes (IFN-β, IFITM3, ISG15, and Mx-2) and (**C**) mouse genes (IFITM3, ISG15, and Mx-2), normalized with mouse Gapdh. (**D**) Immunofluorescence staining for human ISG15 (green) and nuclei (blue) in the liver of hu-mice (original magnification, ×20). Bars in the scatter plots represent the median value. Statistical analysis was performed with 1-way ANOVA and Turkey’s post hoc test; *P < 0.05; ****P < 0.00005.

We have previously reported that persistent pDC activation (27) and IFN-I signaling contribute to the immunopathogenesis of HIV-1 in the absence or presence of cART (28, 29, 41). We thus investigated IFN-I levels in the liver of hu-mice. Compared with the control groups, a significant increase in human IFN-β and ISGs (IFITM3, ISG15, and Mx-2) was detected in the liver of HIV/cART mice (Figure 3, A and B). Intriguingly, there also was higher induction of mouse ISGs (IFITM3, ISG15, and Mx-2) in the livers of HIV/cART mice and a moderate increase of mouse ISG levels in the cART-alone mice (Figure 3C). This was confirmed by immunofluorescence staining of ISG15 showing more ISG15-positive cells in the liver of HIV/cART mice (Figure 3D). We conclude that HIV/cART-induced liver fibrosis is associated with an accumulation of M2-like (CD68^+^/MerTK^+^) macrophages and with activation of IFN-I signaling in the liver of hu-mice.

### IFN-I directly contributes to activation of human HepSCs.

As reported previously, residual IFN signaling persists in HIV-positive humans and hu-mice treated with suppressive cART and contributes to chronic inflammation-associated diseases (2, 28, 41). We hypothesized that residual IFN-I also contributes to HIV/cART-induced liver fibrosis. To test this hypothesis, primary human HepSCs, isolated from healthy donors (negative for HIV, HBV, and HCV), were exposed to IFN-α2a and IFN-β. To better characterize the effect of types of IFN-I on the activation of HepSCs, we first assessed the autoactivation level of HepSCs by comparing HepSCs seeded on plastic plates with those seeded on plates coated with Geltrex (Thermo Fisher Scientific, A1569601).

Compared with plastic plates, HepSCs from Geltrex-coated plates showed lower expression of α-SMA (from day 1 to day 3) and Col.1a1 (day 2) (Supplemental Figure 3A), indicating that Geltrex limited the autoactivation of HepSCs or induced a resting of HepSCs. Moreover, rested HepSCs exposed to TGF-β led to a strong induction of α-SMA (10-fold) and Col.1a1 (4-fold), validating our ex vivo model of HepSC activation (Supplemental Figure 3B). Therefore, all the following experiments were performed with Geltrex-rested HepSCs.

**Figure 4:**
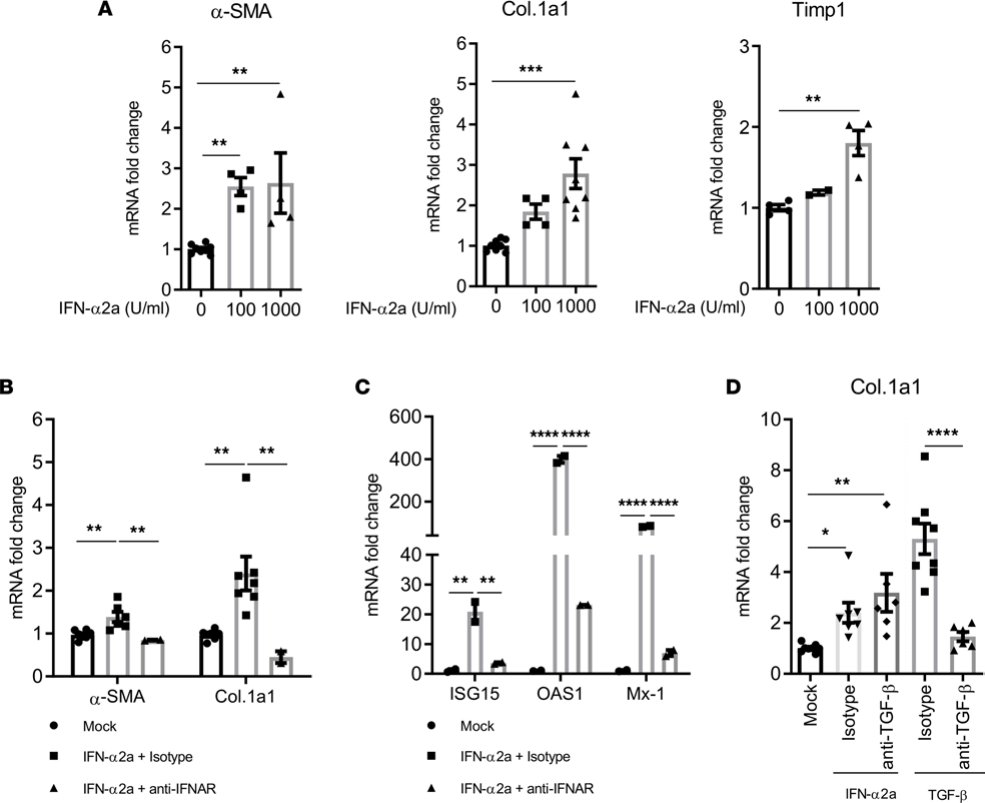
IFN-I can directly activate primary human HepSCs. (**A**) Rested primary human HepSCs were treated with different doses of IFN-α2a. RT-qPCR was performed to measure HepSC-activation genes α-SMA, Col.1a1, and Timp1. (**B** and **C**) Rested HepSCs were treated with IFN-α2a after exposure to anti-IFNAR1 Ab or isotype control. Expression of α-SMA, Col.1a1, ISG15, OAS1, and Mx-1 was detected by RT-qPCR. (**D**) TGF-β is not involved in IFN-α2a–induced HepSC activation. Rested HepSCs were treated with IFN-α2a or TGF-β in the presence of isotype or anti-TGF-β Ab. Expression of Col.1a1 was detected by RT-qPCR. Data were normalized with Gapdh and represent the average of 3 independent experiments; error bars indicate the SEM. Statistical analysis was performed with 1-way ANOVA and Fisher’s LSD test. *P < 0.05; **P < 0.005; ***P < 0.0005; ****P < 0.00005.

Interestingly, in HepSCs exposed to IFN-α2a, an induction of α-SMA (2.8-fold), Col.1a1 (2.8-fold), and Timp1 (1.8-fold) mRNAs was detected (Figure 4A). Similarly, exposure of HepSCs to IFN-β also resulted in a 4.5-fold increase in Col.1a1 transcription (Supplemental Figure 4). To confirm IFN-I signaling was required, blockade of IFN-I receptors with the anti-IFNAR1 Ab prevented IFN-α2a–induced α-SMA and Col.1a1 expression (Figure 4B), and the expression of ISG15, OAS1, and Mx-1 (Figure 4C) in HepSCs. Two different donors of HepSCs were used to confirm these findings.

We next investigated if the capacity of IFN-I to activate HepSCs is TGF-β dependent. To test this, HepSCs were co-exposed for 2 days to IFN-α2a and either the isotype control IgG or anti–TGF-β neutralizing Ab. We detected no difference in the induction of Col.1a1 between HepSCs with the isotype control and anti–TGF-β Ab (Figure 4D). Therefore, our data indicate that IFN-I interacted with the IFNAR receptors and activated HepSCs via a TGF-β-independent mechanism.

### IFN-I and TGF-β synergistically activate HepSCs.

**Figure 5:**
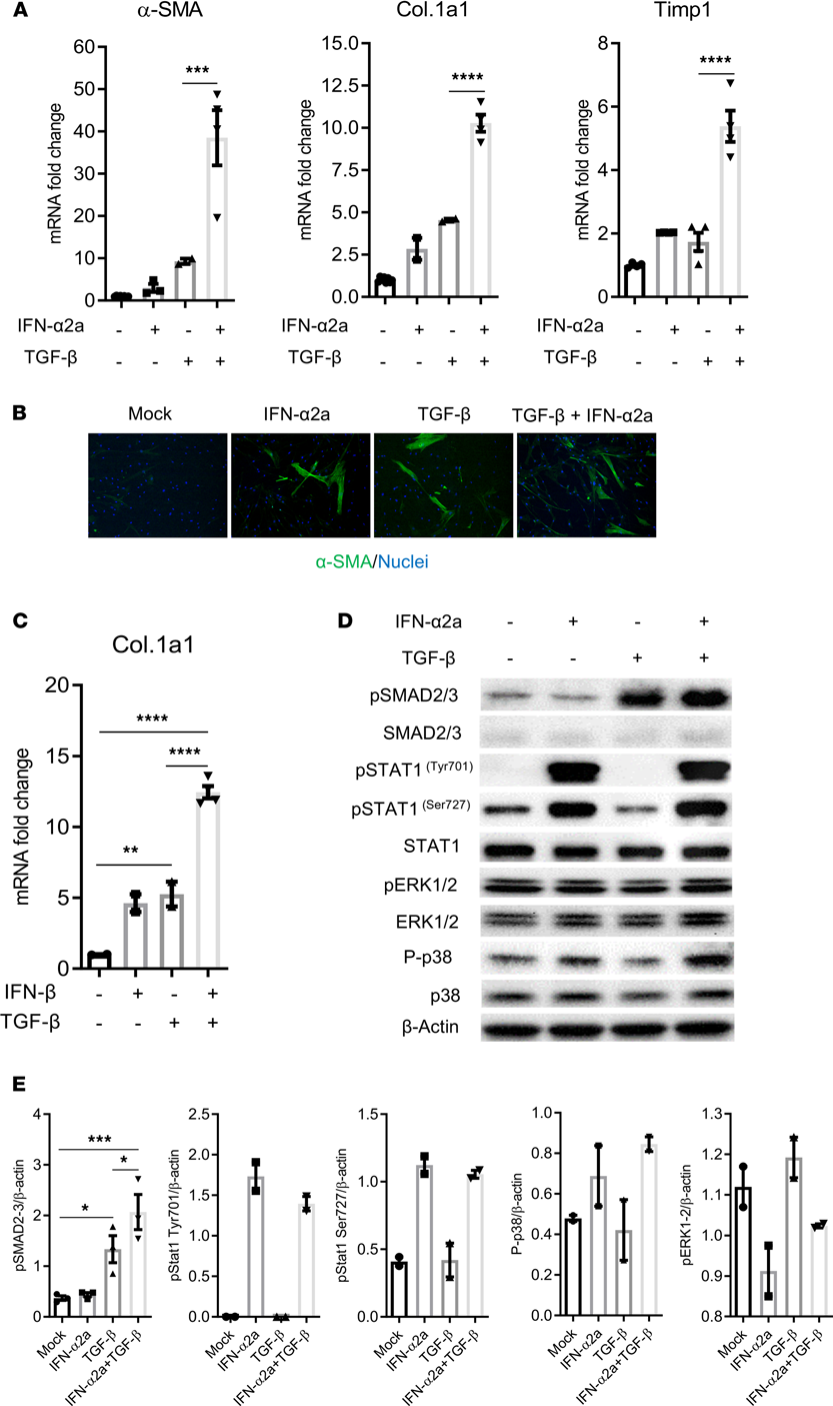
IFN-I and TGF-β synergistically activate primary human HepSCs. (**A** and **B**) Rested HepSCs were treated with IFN-α2a (1000 U/mL) and/or TGF-β (1 ng/mL). (**A**) Expression of α-SMA, Col.1a1, and Timp1 was detected by RT-PCR. (**B**) Immunofluorescence of α-SMA (green) and nuclei (blue) in HepSCs exposed to TGF-β, IFN-α2a, and both (original magnification, ×20). (**C**) Rested HepSCs were treated with IFN-β (100 U/mL) and TGF-β (1 ng/mL). Expression of Col.1a1 was detected by RT-PCR. Data were normalized with Gapdh. (**D** and **E**) IFN-I increase TGF-β–induced activation of phosphorylation of SMAD2/3 in HepSCs treated with IFN-α2a and TGF-β. Rested HepSCs were treated with IFN-I and/or TGF-β for 60 minutes. (**D** and **E**) Immunoblot analysis (**D**) and quantification (**E**) of phosphorylation levels of SMAD2/3, STAT1 (Tyr701 and Ser727), p38 and ERK1/2, and of total SMAD2/3, STAT1, p38, ERK1/2, and β-actin in HepSCs. Histograms represent the average of 2–3 independent experiments; error bars indicate the SEM. Statistical analysis was performed with 1-way ANOVA and Fisher’s LSD test. *P < 0.05; **P < 0.005; ***P < 0.0005; ****P < 0.00005.

We next postulated that the increase of liver fibrosis in HIV/cART mice may be due to a cooperative effect between IFN-I and TGF-β in the activation of HepSCs. Compared with IFN-I and TGF-β alone, which only led to approximately a 2-fold and 8-fold induction of α-SMA, respectively, we observed that exposure to both IFN-α2a and TGF-β resulted in a 30-fold synergistic induction of α-SMA. IFN-α2a and TGF-β also synergistically induced Col.1a1 (10-fold) and Timp1 (5.8-fold) expression (Figure 5A and Supplemental Figure 5) in different experiments. Immunofluorescence staining also showed more α-SMA–positive cells in HepSCs co-exposed to IFN-α2a and TGF-β than single controls (Figure 5B). Similarly, HepSCs exposed to IFN-β and TGF-β also revealed synergistic upregulation of Col.1a1 (12.5-fold), compared with each cytokine alone (Figure 5C). Exposure to IFN-I or TGF-β alone or both did not affect the proliferation of or induce cell death in HepSCs (Supplemental Figure 6) (other data not shown). Together, these results showed a cooperative effect between IFN-I and TGF-β in the activation of HepSCs.

To understand how IFN-I and TGF-β interact to activate HepSCs, we measured the phosphorylation of key components of the Jak/Stat and Smad signaling pathways. As expected, IFN-α2a or TGF-β alone efficiently induced the phosphorylation of Stat1 Tyr701/Ser727 and Smad2/3, respectively. Interestingly, combination of IFN-α2a and TGF-β induced a higher level of phosphorylation of Smad2/3 in HepSCs than of TGF-β alone (Figure 5, D and E). No difference in Stat1 phosphorylation on Tyr701 and Ser727 was observed between cells exposed to IFN-α2a alone and to the combination IFN-α2a and TGF-β (Figure 5, D and E). Moreover, no significant difference in the phosphorylation of ERK1/2 and p38 was detected between different treatment conditions (Figure 5, D and E). Taken together, our results revealed that IFN-I potentiate TGF-β–induced Smad signaling activation, leading to the enhancement of HepSCs activation.

### Blockade of IFN-I signaling prevents HIV-1/cART–induced liver fibrosis.

**Figure 6:**
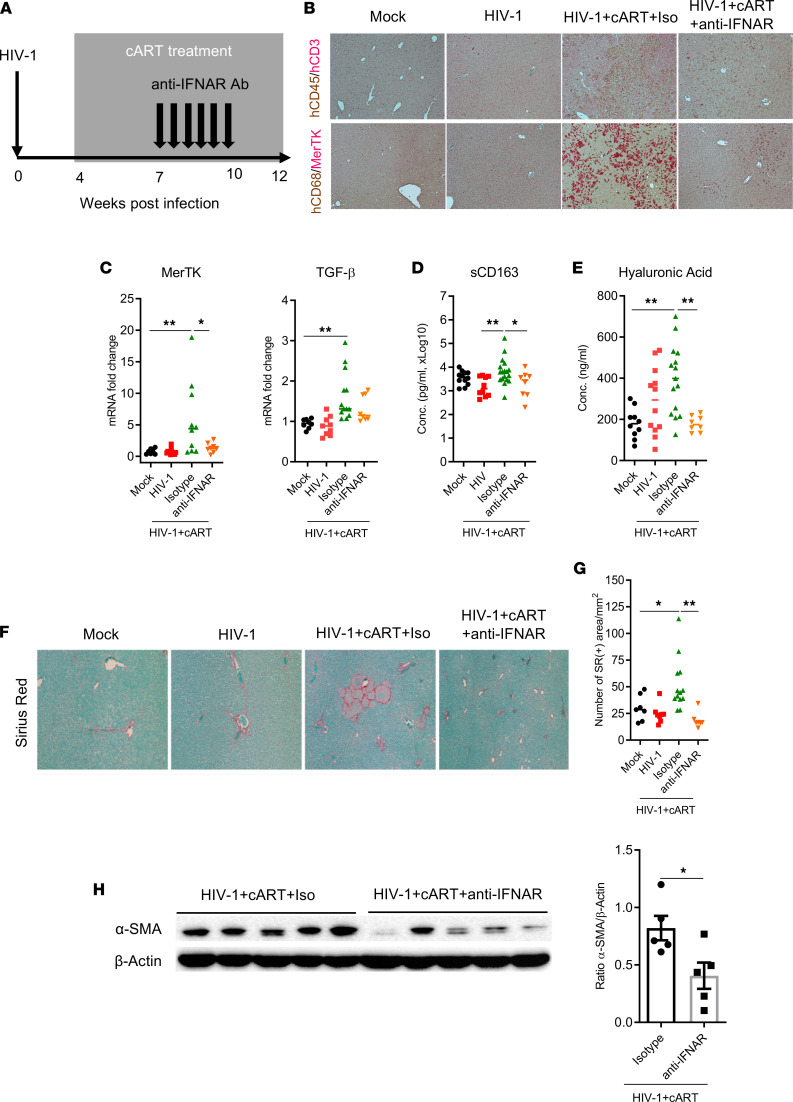
Blockade of IFN-I signaling prevents HIV/cART-induced hepatic accumulation of M2-like macrophages and liver fibrosis or diseases in hu-mice. (**A**) Diagram of experimental design. Blood and liver tissues from mock- and HIV-infected NRG-hu HSC mice treated with cART were analyzed at 12–13 wpi. (**B–D**) Assessment of the anti–IFNAR1 Ab treatment on liver immune infiltration. (**B**) Liver sections were costained for human CD45/CD3 (brown/red), CD68/MerTK (brown/red) by IHC (original magnification, ×20). (**C**) Expression of human MerTK and TGF-β was detected in the liver by RT-qPCR; data were normalized with mouse Gapdh. (**D**) Soluble CD163 levels were measured in the blood of hu-mice by ELISA. (**E**) Quantification of hyaluronic acid in the serum by ELISA. (**F–H**) Decrease of liver fibrosis in animals treated with anti-IFNAR1. (**F**) Liver sections were stained for SR and (**G**) collagen deposition automatically was quantified by deconvolution algorithm analysis. (**H**) Immunoblot and quantification of α-SMA expression in the liver. Histograms represent the SEM; bars in the scatter plots represent the median. Statistical analysis was performed with 1-way ANOVA and Turkey’s post hoc test. *P < 0.05; **P < 0.005. Conc., concentration.

To functionally define the role of IFN-I in liver fibrosis in HIV/cART mice, we treated mice from 7 to 10 wpi with anti–IFNAR1 Ab or an isotype control and euthanized mice at 12–13 wpi (Figure 6A). Interestingly, anti–IFNAR1 Ab treatment reduced the infiltration of human immune cells (CD45^+^/CD3^–^ and CD68^+^/MerTK^+^) in the liver of HIV/cART mice (Figure 6B and Supplemental Figure 7), which was consistent with a decrease in expression of human MerTK and TGF-β mRNAs in the liver (Figure 6C), as well as the soluble CD163 in the blood of HIV/cART mice with administered anti–IFNAR1 Ab (Figure 6D). Consistently, the assessment of hyaluronic acid in the blood, a surrogate marker of liver injury or fibrosis, also showed that anti–IFNAR1 Ab treatment induced a significant reduction of hyaluronic acid level in HIV/cART mice (Figure 6E). Importantly, the quantification of SR staining of collagen in the liver section also showed IFNAR1 blockade significantly reduced collagen deposition in the liver of HIV-infected mice with cART (Figure 6, F and G) and the assessment of liver fibrosis at 12–13 wpi by immunoblot revealed that IFNAR1 blockade significantly reduced the expression levels of α-SMA in the liver of HIV/cART animals (Figure 6H), indicating that IFN-I signaling critically contributes to HIV/cART-induced liver fibrosis. Altogether, our data strongly support that activation of IFN-I signaling in the liver contributes to HIV/cART-induced hepatitis and liver fibrosis in hu-mice.

**Figure 7:**
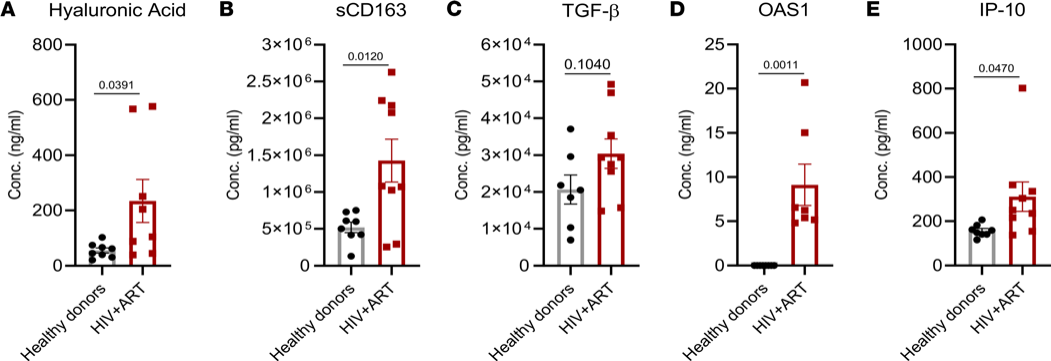
Increase in liver injury, M2-like macrophages, and ISG markers in the blood of PLWH with cART. Liver injury and fibrosis marker (**A**) hyaluronic acid, M2 macrophage markers (**B**) soluble CD163 (sCD163) and (**C**) TGF-β, and IFN-stimulated genes (**D**) OAS1 and (**E**) IP-10 were assessed in plasma samples from HIV-positive individuals with stable cART and healthy individuals, by ELISA. Bars in histogram represent the SEM value. Statistical analysis was performed with an 2-tailed unpaired *t* test. *P* < 0.05 is considered significant. Conc., concentration.

To test if similar inflammatory and liver injury markers are also elevated in PLWH as in hu-mice, we analyzed plasma samples for liver injury or fibrosis and M2-like macrophage markers, and ISGs in the blood of PLWH with stable cART and healthy individuals (Supplemental Table 6). Compared with healthy donors, we found a moderate elevation of ALT levels (Supplemental Table 6) and a significant increase in hyaluronic acid (Figure 7A) in the plasma of PLWH receiving ART. We also detected a significant augmentation in M2 macrophages markers such as human soluble CD163 (sCD163) in PLWH with cART, although plasma levels of TGF-β were similar in both groups (Figure 7, B and C). Interestingly, the plasma levels of the IFN-stimulated genes OAS1 and IP-10 were also significantly elevated in PLWH receiving cART compared with healthy individuals (Figure 7, D and E). Taken together, HIV/cART-induced pathogenic M2-macrophages and activation of IFN signaling may also play a role in increased risk of liver diseases reported in PLWH receiving ART (45–47).

## Discussion

Non-AIDS-defining end-organ diseases are a major issue for PLWH treated with ART; among these diseases, those related to the liver contribute significantly to their morbidity and mortality (1, 2). The virologic and immunologic mechanisms are poorly understood, in part because of a lack of a robust animal model of liver diseases induced by HIV infection and ART. We report here that HIV-1 infection treated with ART leads to hepatitis and liver fibrosis in a humanized mouse model. HIV-1 infection and ART activated both IFN-I and TGF-β signaling pathways in the liver. Mechanistically, HIV-induced IFN-I cooperate with TGF-β to induce the activation of HepSCs. Finally, blocking IFN-I signaling reversed HIV/cART-induced hepatitis and liver fibrosis in hu-mice.

In this study, to investigate the mechanism of liver diseases induced by HIV infection and ART, we used NRG mice reconstituted with human fetal liver–derived CD34^+^ progenitor cells to model HIV-induced liver disease with stable ART as reported in PLWH (28, 30, 31, 41). The hu-mice during the study did not develop graft-versus-host disease symptoms such as conjunctivitis, alopecia, or wasting diseases (data not shown). The key findings in this study were thus not significantly affected by graft-versus-host disease. The ART doses we used are the doses necessary to obtain stable HIV suppression until the undetectable limit in blood and lymphoid tissue in our model. We did not observe any spontaneous death of animals over 20 weeks’ follow-up (28, 30, 31, 41) (other data not shown), and our ART regimen was similar to those reported by other investigators (42–44) (Supplemental Table 1). Interestingly, chronic HIV infection under long-term cART in hu-mice led to liver fibrosis with induction of liver hepatitis and accumulation of MerTK^+^/M2-like macrophages. Similar pathogenic macrophages and liver fibrosis resulting from HBV or HCV have been documented in hu-mice with both human immune system and human hepatocytes or in human patients (33, 34). Therefore, hu-mice engrafted with human fetal liver progenitor cells support long-term HIV-1 infection, respond to cART with stable viral suppression, and develop hepatitis and liver fibrosis. This may provide a robust small-animal model to study virologic and immunologic mechanisms as well as to develop novel therapeutics for liver diseases in PLWH treated with ART.

HIV-1 alone did not induce liver fibrosis in hu-mice (Figure 1 and Supplemental Figures 1 and 8), even though previous reports showed that CXCR4-tropic HIV-1 induces the activation of HepSCs in vitro (48–50). That is probably due to a lack of liver inflammation or of direct interaction between HIV-1 and HepSCs in the liver in vivo. In vitro, HIV exacerbated an HCV-driven profibrogenic program in a coculture system with hepatocytes and HepSC lines through ROS, NF-κB, and TGF-β1 up-regulation, indicating HIV-1 may contribute to the fast progression of liver fibrosis initiated by HCV infection (11). In some hu-mice, we observed that cART alone slightly induced expression of liver injury and fibrosis markers (Figure 1 and Supplemental Figure 1). We speculate that, due to its possible low hepatotoxicity, cART-induced liver injury served as an initiator of liver fibrotic process associated with liver infiltration of HIV-induced inflammatory M2-like cells and elevated IFN-I signaling.

We have previously reported that HIV infection activates IFN-I signaling pathways that contribute to HIV-induced immunopathology in the spleen and bone marrow of hu-mice (28, 30, 41). During HIV-1 infection, IFN-I (via pDCs) are induced and contribute to HIV-induced immune suppression and lymphoid tissue injury, but not in the liver. After the ART regimen, HIV replication is suppressed and becomes undetectable in blood by PCR or in lymphoid tissues by HIV gag/p24. However, animals develop hepatitis with hepatic accumulation of macrophages with M2 phenotype expressing CD163, MerTK, and TGF-β. That finding was consistent with recent data showing that MerTK^+^ macrophages activate HepSCs and induce nonalcoholic steatohepatitis fibrosis through upregulation of TGF-β production (51). In the present study, we observed that cART alone triggered an activation of IFN-I signaling in the liver. Interesting, tenofovir disoproxil fumarate and emtricitabine used for HIV treatment have been recently reported to stimulate an IFN-I/III signature in the gut independently of HIV infection (52). The capacity of ART alone in hu-mice to trigger a low level of mouse IFN-I signaling could be explained by ART-induced liver injury. Importantly, we observed that the combination of HIV and cART induced higher activation of IFN-β and ISGs in the liver of hu-mice, which may cause chronic immune activation in the liver despite HIV suppression by cART. Notably, the HIV-induced dysfunction of gut barrier and microbial translocation that persist after stable cART may also contribute to persistent IFN-I induction and tissue injury (4, 53). Investigation of gut microbial translocation in the future will provide additional insights on how HIV/cART induce liver fibrosis in the humanized mouse model.

We then tested if IFN-I can directly or indirectly, or via cooperation with TGF-β, affect the activation of HepSCs (or liver fibrosis). Using primary human HepSCs, we established in vitro conditions of resting HepSCs that responded to fibrogenic activators such as TGF-β (54–58). Interestingly, IFN-I directly activated HepSCs in a dose-dependent fashion. Blockade of IFN-I signaling, but not of TGF-β, completely abrogated its activity to activate fibrotic genes in HepSCs (Figure 4). Therefore, IFN-I signaling directly contributed to activation of HepSCs. When combined with TGF-β, a synergistic activation of HepSCs was detected, and IFN-I signaling cooperated with TGF-β/Smad signaling by enhancing the phosphorylation of SMAD2/3, leading to higher activation of HepSCs (Figure 5). Thus, the residual IFN-I signaling and inflammation associated with HIV/cART in the liver synergizes with TGF-β to enhance HepSC activation and liver fibrosis. The exact mechanism by which IFN-I interacts with TGF-β to cooperatively activate SMAD2/3 in HepSC is not clearly defined yet but will provide critical insights on how HIV-induced IFN-I contributes to liver fibrosis. SIRT3 signaling has been reported with antifibrotic and antiinflammatory activity (59–61). It is possible that IFN-I and TGF-β may cooperatively reduce SIRT3 signaling, leading to enhancement of HepSC activation and fibrosis.

Finally, when IFN-I signaling was blocked with an IFNAR1 mAb in HIV/cART mice (28, 41), it efficiently prevented the development of liver diseases, including reduced pathogenic macrophages and resolved fibrosis in the liver (Figure 6). These data indicate that HIV-induced IFN-I signaling plays an important role in liver diseases related to HIV/cART and that blocking IFN-I signaling in PLWH treated with cART may provide a novel strategy to treat non-AIDS end-organ diseases such as liver fibrosis (28, 62). These findings shed light on the development of therapeutics of HIV/cART-induced liver diseases by targeting IFN-I production or its signaling pathway.

Humanized mouse models clearly have limitations (63) that limit the mouse model in terms of fully investigating the exact human liver disease progression. They may contribute to the heterogeneity among HIV/cART animals (63). First, the human immunity developed in hu-mice is not fully functional, as in immunocompetent hosts (64, 65). With human CD34^+^ hematopoietic stem cells (HSCs) from human fetal liver, they generally are not efficiently engrafted with mature human liver cells such as hepatocytes and HepSCs (33, 66). Human IFN-I signaling activation was induced in the liver of HIV/cART mice and may lead to induction of ISGs in mouse liver and stellate cells. We showed that HIV/cART-induced liver disease is mediated by TGF-β and IFN-I signaling pathways. Both mouse and human cells may respond to these cytokines. It is possible that HIV/cART-induced human cytokines such as TGF-β and IFN-I activated mouse HepSCs to contribute to liver fibrosis.

There are currently more than 30 antiretroviral drugs, categorized in 8 mechanistic classes, approved by the FDA for the treatment of HIV infection (67). The commonly used drugs are nucleoside and nucleotide reverse transcriptase inhibitors (e.g., emtricitabine; lamivudine; tenofovir disoproxil fumarate) in combination with integrase inhibitors (i.e., raltegravir). ART has significantly improved the lifespan of PLWH. Because most drugs are metabolized by the liver, even low hepatotoxicity becomes an important clinical issue for PLWH with long-term cART (45–47). Therefore, monitoring PLWH with stable cART for potential emergence of liver injury and disease should continue to be of clinical importance.

We next studied liver injury and fibrosis and M2-like macrophage markers in plasma samples from PLWH with stable cART and from healthy individuals. Consistent with our findings in hu-mice, we detected a significant increase in liver injury or fibrosis and M2-like macrophage markers in PLWH with ART compared with healthy individuals. PLWH with cART also had higher plasma levels of ISGs despite cART-induced HIV suppression. Although these data would need to be confirmed in liver biopsy specimens from patients with HIV treated with ART, our findings showed that HIV-infected hu-mice with cART recapitulated human-like liver disease and this model could be a powerful tool to dissect the mechanisms of some aspects of non-AIDS-defining end-organ diseases in PLWH with stable ART.

## Methods

See the Supplemental Methods for additional information.

### Construction of NRG-hu HSC mice.

All animal studies were approved by the University of North Carolina and University of Maryland IACUCs. All mice were housed and bred at the University of North Carolina at Chapel Hill. Human CD34^+^ progenitor cells were isolated as described previously (28, 41). Briefly, human CD34^+^ progenitor cells were isolated from 16- to 20-week-old fetal liver tissues after digestion with Liver Digest Medium (Invitrogen). The suspension was filtered through a 70 μm cell strainer (BD Falcon) and was centrifuged at 150*g* for 5 minutes to isolate mononuclear cells by Ficoll. CD34^+^ progenitor cells were selected with the CD34^+^ magnetic activated cell-sorting kit and 0.2 × 10^6^ cells injected into the liver of NRG (NOD Rag2^–/–^γc-null) mice 2–6 days old (Jackson Laboratory) after irradiation at 200 cGy. At 12 weeks after transplantation, human immune cell reconstitution was determined by flow cytometry.

### HIV-1 infection in hu-mice and cART.

The R5 tropic strain of HIV-1 (JR-CSF) was generated by transfection of 293T cells with plasmid containing full-length HIV-1 (JR-CSF) genome. Hu-mice with stable human leukocyte reconstitution were infected with HIV-1 (JR-CSF; 10 ng of p24 or 3000 infectious units per mouse) through the retro-orbital route. Hu-mice infected with 293T supernatant were used as mock control groups. HIV-1 viral load was quantified in plasma or serum of Hu-mice, as reported previously (28, 41).

Individual antiretroviral drugs were applied to the animals’ food. Final concentrations of drugs in the food were 4800 mg/kg raltegravir, 1560 mg/kg tenofovir disoproxil, and 1040 mg/kg emtricitabine); the estimated daily drug doses were 768 mg/kg raltegravir, 250 mg/kg tenofovir disoproxil, and 166 mg/kg emtricitabine. Animals were exposed to cART from 4 weeks after HIV-1 infection until the end of the experiment, at week 12 or 13.

### IFNAR1-blocking Ab treatments.

An α-IFNAR1 mAb that specifically binds to human IFNAR1 and inhibits human IFN-I activity was developed (28). From 7 to 10 weeks after infection, the cART-treated, HIV-1-infected mice were injected with α-IFNAR1 Ab or isotype control mIgG2a Ab twice a week (400 μg/mouse) at the first injection and 200 μg/mouse for the following treatments. Cohorts of mice were randomized into different treatment groups by level of HIV-1 RNA in plasma.

### Patients.

Plasmas samples from healthy donors (*n* = 8) and HIV-positive individuals with stable cART (*n* = 9) were obtained from the clinic at Institute of Human Virology, University of Maryland School of Medicine. Samples were collected from HIV-positive individuals with cART at about 6 months after HIV was suppressed to undetectable level (<50 HIV-1 genomes/mL). Samples from the control group were obtained from healthy donors who were negative for HIV, HBV, and HCV and had no reported health issues. The details of donor clinical characteristics are summarized in Supplemental Table 6.

### Data transparency statement.

Data, analytic methods, and study materials will be made available to other researchers.

### Statistics.

Statistical significance was assessed by 2-tailed unpaired Student’s *t* tests (used to analyzed means in normally distributed populations) or 1- and 2-way ANOVA (used to analyze ≥3 variables), using Prism 9 (GraphPad Software).

### Study approval.

All experiments in which animals and human samples were used were conducted according to guidelines for the housing and care of laboratory animals and in accordance with protocols approved by the IACUC and IRB at the University of North Carolina at Chapel Hill and at University of Maryland School of Medicine (Baltimore). Human fetal liver and thymus tissues (gestational age 16–19 weeks) were obtained from elective or medically indicated termination of pregnancy through a nonprofit intermediary working with outpatient clinics (Advanced Bioscience Resources). Informed consent of the maternal donors was obtained in all cases, under regulation governing the clinic. The project was reviewed by the University’s Office of Human Research Ethics, which has determined that this submission does not constitute human subjects research as defined under federal regulations [45 CFR 46.102 (d or f) and 21 CFR 56.102(c) (e) (l)]. Human plasma samples were collected from individuals who gave written informed consent prior to participation in the study approved by the IRB at University of Maryland School of Medicine (Baltimore) (68, 69).

## Author contributions

LS and JA conceived of the study and its design; JA, KN, XZ, GL, MF, and LC contributed to acquisition of data and conducted statistical analysis; SK and EW provided healthy and HIV/cART plasma samples; JA, KN, and LS conducted the data analysis and interpretation; JA and LS drafted the manuscript; LC and LS critically revised the manuscript; and LS obtained funding and supervised the study.

## Supplementary Material

Supplemental data

## Version 1 05/31/2022

In-Press Preview

## Version 2 07/08/2022

Electronic publication
