# Supplementary material for: Type I interferons and TGF-β cooperate to induce liver fibrosis during HIV-1 infection under antiretroviral therapy
Source: JCI Insight. 2022 Jul 8;7(13):e152738. doi: 10.1172/jci.insight.152738 (PMC9310524; doi:10.1172/jci.insight.152738)
Supplement: Supplemental data [file jciinsight-7-152738-s197.pdf]

Type I interferons and TGF-β cooperate to induce liver fibrosis during HIV-1 infection under antiretroviral therapy

James Ahodantin[1,2], Kouki Nio[2,4], Masaya Funaki[1,2], Xuguang Zhai[2,5], Eleanor Wilson[3], Shyamasundaran Kottilil[3], Liang Cheng[2,6], Guangming Li[1,2] and Lishan Su[1,2]

[1]Division of Virology, Pathogenesis and Cancer, Institute of Human Virology, Departments of Pharmacology, Microbiology and Immunology, University of Maryland School of Medicine, Baltimore, MD 21201, USA, [2]Lineberger Comprehensive Cancer Center, [2]Department of Microbiology and Immunology, The University of North Carolina at Chapel Hill, Chapel Hill, NC 27599, USA. [3]Division of Clinical Care & Research, Institute of Human Virology, Department of Medicine, University of Maryland School of Medicine, Baltimore, MD 21201, USA.

[4]Current address: Department of Gastroenterology, Kanazawa University Hospital, Kanazawa, Ishikawa 920-8641, Japan

[5]Current address: Department of Biochemistry and Molecular Biology, Medical College, Nantong University, Nantong, Jiangsu, China

[6]Current address: Frontier Science Center for Immunology and Metabolism, Medical Research Institute, Wuhan University, Wuhan, China

*To whom correspondence should be addressed:

Lishan Su, PhD

725 West Lombard St., N362

Baltimore, MD 21201, USA

lsu@ihv.umaryland.edu

Office Tel.: 410-706-7878

Supplemental materials and methods:

Histology and Immuno-histochemistry/fluorescence staining.

For IHC, paraffin-embedded formalin-fixed liver sections (5 µm) from Hu-mice were stained with hematoxylin and eosin (H&E), Sirius red, or with the following primary antibodies after antigen retrieval: anti-human CD45 (1:2, #IS75130-2), CD3 (1:100, #A0452), CD68 (1:100, #M081401-2), all from Dako; CD163 (1:100, #ab87099) and MerTK (1:100, #ab52968) from Abcam. Then incubated with the secondary antibodies and revealed following the manufacturer's instructions (Dako and IHC World). For immunofluorescence staining, liver sections and HepSCs were stained for CD68 (1:100, #M081401-2), MerTK (1:100, #ab52968), ISG15 (1:200, Proteintech #15981-1-AP) and anti α-SMA (1:100, ab7817) respectively then detected by incubating with a secondary anti-mouse and anti-rabbit conjugated with Alexa Fluor 488 (1:500) and Alexa Fluor 555 (1:500) (Invitrogen) respectively. Nuclei were counterstained with Hoechst 33342 (Sigma). Sirius Red stained slides were digitally imaged at 20x objective in the Aperio ScanScope XT (Leica). Scanned slides have been divided into three groups (normal, light, and pale) based on the appearance of the green counterstain defining non-collagen tissue. Aperio color deconvolution algorithm v9 customized for each group of slides was used to separate collagen positive area (red) from the non-collagen (green), calculate size and percentage of the positive stain at each staining intensity (strong, medium, week, negative) and generate the Score (0-300). The Score (0-300) = (3 x % Strong Positive) + (2 x % Medium Positive) + (1 x % Weak Positive). Input RGB values of the green stain were appropriately adjusted in the algorithms used for each group as well as the weak positive threshold. To exclude positively stained large blood vessels from the analysis the Genie tissue classifier was developed and added to the algorithm.

Culture and activation of primary human hepatic stellate cells.

Primary human hepatic stellate cells (HepSCs) from 2 different donors (HepSC-74 and HepSC-75), were obtained from iXCells Biotechnologies company and expanded

following the manufacturer's instructions. All experiments were performed at passage 3. HepSCs were rested with Geltrex-coated plates for one day prior exposure to recombinant human TGF-β (0.1-5 ng/ml, reference #240-B R&D Systems) and/or recombinant human type I interferons (IFN-α2a and IFN-β, 10-1000 U/ml; references #11100-1 and #11410-2 PBL Science respectively) in DMEM without FBS for one hour or two days. For IFN-α/β receptor (IFNAR) blockade, rested HepSCs were incubated for one hour with either 10 ug/ml of anti-IFNAR antibody or isotype control (reference #MAB003 R&D Systems). For TGF-β neutralization experiment, 1 ng/ml of TGF-β were incubated for an hour with 5 ug/ml of either anti- TGF-β antibody (reference # MAB1835-500 R&D Systems) or isotype control before HepSCs exposure.

Immunoblot blot.

Total proteins were extracted from fresh liver tissues or HepSCs using a RIPA buffer (Thermo Scientific) with protease and phosphatase inhibitor cocktail (Pierce). Protein extracts were resolved on SDS-PAGE (12%), transferred onto a nitrocellulose membrane and incubated with the following primary antibodies at 1:250 to 1:1000 dilution: phospho-SMAD2/3 (#8828) , phospho-STAT1 Tyr701 (#9167), phospho-STAT1 Ser727 (#8826), phospho-p38 (#4511) and phospho-ERK1/2 (#9101) and total SMAD2/3 (#8685), STAT1 (#14994), p38 (#9212), ERK1/2 (#4695), all from Cell Signal; α-SMA (1:100, ab7817), CCND1 (#ab16663), CCNA2 (#ab181591) from Abcam and β-actin (#A3854, Sigma/Millipore); secondary antibodies were either anti-mouse or anti-rabbit coupled with horseradish peroxidase, and bands were revealed using the ECL (Millipore and Invitrogen) with BioRad CCD camera.

Quantification of secreted proteins by ELISA.

Soluble human CD163 (sCD163, DuoSet # DY1607 from R&D Systems), ALT (#XPEM0829 from XpressBio), Hyaluronic acid (HA, Echelon Biosciences Inc), TGF-β (RND #DB100C), OAS1 (LSBio #LS-F31934-1) and IP-10 (RND #DIP100) secretions

were detected in plasmas and analyzed by ELISA following the manufacturer's instructions.

mRNAs expression analysis.

Total RNA was extracted from cells (RNeasy Plus Kit, QIAGEN) and from liver tissues using QIAzol Lysis Reagent and gDNA Eliminator Solution (RNeasy Plus Universal Kits, QIAGEN), and quantified using Nanodrop. 0.2-2 µg of total RNAs were used for cDNA preparation by reverse transcription with random primers and SuperScript III (Invitrogen), according to the manufacturer's instructions. Two µl of diluted (1:10) cDNA were used for quantification with the Power SyBR Green PCR Master Mix (Applied Biosystems) on the QuantStudio 6 flex (Applied Biosystems, Foster City, CA), using specific primers (Table S3 and S4). The comparative Ct method was used for the analysis of real-time PCR data(1, 2). Data were normalized using human or mouse Gapdh as the housekeeping gene and expressed as the relative mRNA level compared to the controls.

Table S1. Comparison of ART doses for HIV treatment in food pellets (mg/kg).

Table S2. Summary of NRG-hu mouse cohorts 1, 2 and 3 defined by HSC donors and individual mice.

Table S3. Human oligonucleotides/PCR primers used in the study.

Table S4. Mouse oligonucleotides/PCR primers used in the study.

Table S5. Summary of NRG-hu mouse cohorts 4 and 5, and individual mice

Table S6. Summary of Clinical information of Human cohort and individual specimens

Figure S1. Assessment of liver injury and fibrosis during HIV infection and ART regimen. (**A**) Microscopic analysis of fibrosis incidence (SR^+ vs SR^-) in the liver of humanized mice. (**B**) Detection by RT-qPCR of fibrosis genes (mouse α-SMA, Col.7a1, Timp1 and MMP-13) in the liver of humanized mice infected with HIV-1 and treated with

cART and their littermate controls. Data were normalized with mouse Gapdh. Bars indicate the median. Statistical analysis was performed with one-way Anova and Turkey test; *$p < 0.05$; **$p < 0.005$.

Figure S2. Characterization of liver infiltrated human immune cells. (**A**) Immunofluorescence validation for anti-MerTK antibody versus isotype control (green) using liver sections from HIV/cART mice. Illustration of single staining and their merge. Co-staining with isotype antibody (green) and nuclei (bleu) by immunofluorescence in liver sections of HIV/cART mice. Images were acquired with 20x magnification lens.

Figure S3. Optimization of resting and activating primary human hepatic stellate Cells (HepSCs). RT-qPCR analysis of HepSC activation genes (α-SMA and Col.1a1). (**A**) Geltrex prevents the auto-activation of human primary hepatic stellate cells in vitro (α-SMA and Col.1a1). (**B**) TGF-β activates HepSCs in a dose-dependent manner (α-SMA and Col.1a1). Data were normalized with Gapdh. Histograms represent the average of different independent experiments. Error bars indicate the SEM. Statistical analysis was performed with one-way Anova and Fisher's LSD test; *$p < 0.05$; **$p < 0.005$; ***$p < 0.0005$; ****$p < 0.00005$.

Figure S4. IFN-β induces activation of primary human hepatic stellate cells. Dose-dependent activation of HepSCs by IFN-β; (**A**) RT-qPCR analysis of hepatic stellate cell activation genes α-SMA and Col.1a1. Histograms represent the average of different independent experiments. Error bars indicate the SEM. Statistical analysis was performed with one-way Anova and Fisher's LSD test; *$p < 0.05$; **$p < 0.005$.

Figure S5. Synergistic effect of IFN-α2a and TGF-β on activation of human HepSCs from Donor #2; RT-qPCR analysis of hepatic stellate cell activation genes (**A**) α-SMA and (**B**) Col.1a1. Histograms represent the average of different independent experiments.

Error bars indicate the SEM. Statistical analysis was performed with one-way Anova and Fisher's LSD test; *p < 0.05; **p < 0.005.

Figure S6. Effects of IFN-α2a and TGF-β on genes involved in proliferation/apoptosis of primary human hepatic stellate Cells. (**A**) RT-qPCR (CCNA2, CCND1 and PCNA) and (**B**) Immunoblot (CCNA2 and CCND1) analysis of cell proliferation markers. Histograms represent the average of different independent experiments. Error bars indicate the SEM. Statistical analysis was performed with one-way Anova and Fisher's LSD test.

Figure S7. Blocking of IFN-α/β receptor prevents the accumulation of human M2-like macrophages in the liver of HIV-infected mice under cART. (**A**) Immunohistochemistry staining for human CD45, CD68 and CD163 in the liver of humanized mice infected with HIV and treated with cART and their littermate controls.

Figure S8. Confirmation of IFN-I blockade alleviates HIV/ART induced liver fibrosis in humanized mice in two supplemental cohorts 4 and 5. For post-HIV infection, animals were treated with cART for 9 weeks and blood and liver samples collected at week 13 for analysis. Elisa detection of (**A**) ALT and (**B**) Hyaluronic acid (HA) in plasmas at week 13. Quantification of fibrosis markers (**C**) α-SMA and (**D**) TGF-β by RT-qPCR in the liver at week 13. Bars in the scatter plots represent the median value. Statistical analysis was performed with one-way ANOVA and Turkey’s post-hoc test; *p < 0.05; **p < 0.005; ***p < 0.0005.

REFERENCES

1. Ahodantin J, Bou-Nader M, Cordier C, Megret J, Soussan P, Desdouets C, et al. Hepatitis B virus X protein promotes DNA damage propagation through disruption of liver polyploidization and enhances hepatocellular carcinoma initiation. *Oncogene.* 2019;38(14):2645-57.
2. Ahodantin J, Lekbaby B, Bou Nader M, Soussan P, and Kremsdorf D. Hepatitis B virus X protein enhances the development of liver fibrosis and the expression of genes associated with epithelial-mesenchymal transitions and tumor progenitor cells. *Carcinogenesis.* 2020;41(3):358-67.

Table S1. Comparison of ART doses for HIV treatment in food pellets (mg/kg)

	Current Study	Halper-Stromberg A, et al. Cell 2014	Lavender KJ, et al. AIDS 2018	Nischang M, et al. PlosOne 2012
Tenofovir Disoproxil Fumarate (TDF)	1,560	720	720	500
Emtricitabine (FTC)	1,040	520	520	N/A
Raltegravir (RAL)	4,800	4800	4800	N/A
Lamivudine (3TC)	N/A	N/A	N/A	500
Azidothymidine (AZT)	N/A	N/A	N/A	500
Ritonavir (RTV)	N/A	N/A	N/A	1000

- N/A : Not Applicable

Table S2. Summary of NRG-hu mouse cohorts and individual mice

HSC donors	Mouse ID	%hCD45	Status	Duration of cART	HIV titer (xLog_{10} c/ml) at termination
1	111	40.9	Mock	NA	UD
1	112	77.9	Mock	NA	UD
1	113	76.8	Mock	NA	UD
1	114	82.3	Mock	NA	UD
1	115	84.7	Mock	NA	UD
1	116	75.0	HIV-1	NA	5.45
1	117	70.1	HIV-1	NA	5.82
1	118	66.7	HIV-1	NA	4.94
1	119	68.3	HIV-1	NA	5.16
1	124	50.3	HIV-1	NA	6.34
1	125	46.7	HIV-1	NA	5.21
1	127	38.2	HIV-1	NA	5.17
1	120	52.4	HIV-1+cART	8w	UD
1	121	56.3	HIV-1+cART	8w	UD
1	122	61.0	HIV-1+cART	8w	UD
1	123	48.0	HIV-1+cART	8w	UD
1	128	67.3	HIV-1+cART	8w	UD
1	129	72.2	HIV-1+cART	8w	UD
1	130	75.7	HIV-1+cART	8w	UD
2	50	92.8	Mock	NA	UD
2	51	93.6	Mock	NA	UD
2	52	90.9	HIV-1+cART+Iso	8w	UD
2	53	88.2	HIV-1+cART+Iso	8w	UD
2	58	84.9	HIV-1+cART+Iso	8w	UD
2	59	86.2	HIV-1+cART+Iso	8w	UD
2	60	91.5	HIV-1+cART+Iso	8w	UD
2	61	78.0	HIV-1+cART+Iso	8w	UD
2	62	83.8	HIV-1+cART+Iso	8w	UD
3	3707	71.0	Mock	NA	UD
3	3708	81.2	Mock	NA	UD
3	3709	83.1	Mock	NA	UD
3	3710	61.7	Mock	NA	UD
3	3698	42.1	cART	9w	UD
3	3700	75.6	cART	9w	UD
3	3701	62.1	cART	9w	UD
3	3711	68.1	cART	9w	UD
3	3712	47.2	cART	9w	UD
3	3688	76.6	HIV-1	NA	7
3	3689	81.4	HIV-1	NA	8
3	3690	78.1	HIV-1	NA	7
3	3691	86.7	HIV-1	NA	7
3	3692	83.1	HIV-1	NA	7
3	3683	67.5	HIV-1+cART+Iso	9w	UD
3	3684	62.2	HIV-1+cART+Iso	9w	UD
3	3685	76.3	HIV-1+cART+Iso	9w	UD
3	3686	56.8	HIV-1+cART+Iso	9w	UD
3	3693	51.5	HIV-1+cART+Iso	9w	UD
3	3694	72.3	HIV-1+cART+Iso	9w	UD
3	3695	88.5	HIV-1+cART+anti-IFNAR	9w	UD
3	3696	81.4	HIV-1+cART+anti-IFNAR	9w	UD
3	3697	75.6	HIV-1+cART+anti-IFNAR	9w	UD
3	3702	83.7	HIV-1+cART+anti-IFNAR	9w	UD
3	3703	83.4	HIV-1+cART+anti-IFNAR	9w	UD
3	3704	80.6	HIV-1+cART+anti-IFNAR	9w	UD
3	3705	87.4	HIV-1+cART+anti-IFNAR	9w	UD
3	3706	83.5	HIV-1+cART+anti-IFNAR	9w	UD

cART : combined Anti-Retroviral Therapy, IFNAR : Interferon alpha/beta Receptor, W : Week, N/A : Not Applicable, U/D : Undetectable

Table S3. Human oligonucleotides/PCR primers used in the study

Primers ID	Forward	Reverse
TGF-β	GACATCAACGGGTTCACTACCG	AGAAGCAGGAAAGGCCGGTT
α-sma	GCCAAGCACTGTCAGGAATC	TTGTCACACACCAAGGCAGT
Gapdh	GGAGTCAACGGATTTGGT	AAGATGGTGATGGGATTTCCA
CD163	GGGCTAATTCCAGTGCAGGT	GCTGACTCATTCCCACGACA
COL.1a1	TCTGGCGCTCCCATGGCTCT	GCCCTGCGGCACAAGGGATT
ISG15	CGCAGATCACCCAGAAGATCG	TTCGTCGCATTTGTCCACCA
IFITM3	ATGTCGTCTGGTCCCTGTTC	GTCATGAGGATGCCCAGAAT
Mx-1	GGTGGTCCCCAGTAATGTGG	CGTCAAGATTCCGATGGTCCT
Mx-2	CAGAGGCAGCGGAATCGTAA	TGAAGCTCTAGCTCGGTGTTC
IFN-β	GTGCCTGGACCATAGTCAGAGTGG	TGTCCAGTCCCAGAGGCACAGG
MerTK	AATGACAAAGGGCTGACCGT	TGTGCAGTGCTGTTACGGAT
CCNA2	IDT DNA ref. Hs.PT.56a.4535284	
CCND1	IDT DNA ref. Hs.PT.56a.4930170	
PCNA	IDT DNA ref. Hs.PT.58.4761611	

Table S4. Mouse oligonucleotides/PCR primers used in the study

Primers ID	Forward	Reverse
α-sma	GAGACTCTCTTCCAGCCATCT	CCTGACAGGACGTTGTTAGC
ISG15	AAGCAGCCAGAAGCAGACTC	GTGACGGACACCAGGAAATC
OAS1	GGCTGAAGAGGCTGATGTGT	CAGTTCTCCTCCACCTGCTC
Mx-2	GTGGCAGAGGGAGAATGTCG	TAAAACAGCATAACCTTTTGCGA
IFITM3	GAGGATTCCGACTTCCGGTC	TGTTACACCTGCGTGTAGGG
Gapdh	AGACGGCCGCATCTTCTTGTGCA	GCCCAATACGGCCAAATCCGTTC
Col.7a1	IDT DNA ref. Mm.PT.58.32041766	
Timp1	IDT DNA ref. Mm.PT.58.30682575	
MMP-13	IDT DNA ref. Mm.PT.58.42286812	

Table S5. Summary of NRG-hu mouse cohorts 4 and 5, and individual mice

Cohort	Mouse ID	Status	%hCD45	Duration of cART	HIV titer (xLog10 c/ml) at termination
4	1986	Mock	76.8	N/A	UD
4	1987	Mock	82.3	N/A	UD
4	1988	Mock	84.7	N/A	UD
4	1989	HIV-1	75	N/A	7.5
4	1990	HIV-1	70.2	N/A	6.9
4	1991	HIV-1	68.8	N/A	7.1
4	1993	HIV-1+cART+Iso	83.7	9 w	UD
4	1995	HIV-1+cART+Iso	78.7	9 w	UD
4	1996	HIV-1+cART+Iso	84.1	9 w	UD
4	1997	HIV-1+cART+Iso	74.2	9 w	UD
4	1998	HIV-1+cART+anti-IFNAR	81	9 w	UD
4	1999	HIV-1+cART+anti-IFNAR	70.9	9 w	UD
4	2000	HIV-1+cART+anti-IFNAR	69.4	9 w	UD
4	2001	HIV-1+cART+anti-IFNAR	86.3	9 w	UD
4	2002	HIV-1+cART+anti-IFNAR	74.5	9 w	UD
5	2457	Mock	61.7	N/A	UD
5	2458	Mock	42.1	N/A	UD
5	2382	HIV-1	75.6	N/A	6.1
5	2390	HIV-1	62.1	N/A	5.2
5	2403	HIV-1	68.1	N/A	4.9
5	2392	HIV-1+cART+Iso	47.2	9 w	UD
5	2396	HIV-1+cART+Iso	45.6	9 w	UD
5	2399	HIV-1+cART+Iso	34.1	9 w	UD
5	2400	HIV-1+cART+Iso	40.9	9 w	UD
5	2376	HIV-1+cART+anti-IFNAR	47.4	9 w	UD
5	2377	HIV-1+cART+anti-IFNAR	36.6	9 w	UD
5	2378	HIV-1+cART+anti-IFNAR	29.3	9 w	UD
5	2384	HIV-1+cART+anti-IFNAR	36.9	9 w	UD

cART : combined Anti-Retroviral Therapy, IFNAR : Interferon alpha/beta Receptor, W : Week, N/A : Not Applicable, U/D : Undetectable

Table S6. Summary of Clinical information of Human cohort and individual specimens

				Antiretroviral therapy (ART)		
Specimens ID	Age (years)	Gender	HIV status	Combination	Duration (months)	ALT (U/L)
RPS009	52	Male	Positive	Abacavir/dolutegravir/lamivudine	>=6	43.79
RUM031	52	Male	Positive	Darunavir, Truvada, Ritonavir	>=6	44.18
RUM047	60	Male	Positive	Abacavir/Lamivudine, Darunavir, Ritonavir	>=6	11.63
RUMCHB	54	Female	Positive	Epzicom, Isentress	>=6	13.03
RUMMNC	43	Male	Positive	Darunavir, Truvada, Ritonavir	>=6	16.37
RUMTES	57	Male	Positive	Triumeq	>=6	75.48
RWJ006	58	Female	Positive	Odefsey (had been switched from Complera <30 days prior to screening)	>=6	23.03
RWJ007	63	Female	Positive	Descovy, Raltegravir	>=6	24.62
RWJ008	65	Male	Positive	Emtricitabine and Tenofovir, Isentress	>=6	12.74
BH001	33	Male	Negative	N/A	N/A	11.58
KL002	52	Male	Negative	N/A	N/A	17.55
AA003	35	Male	Negative	N/A	N/A	13.46
AK004	37	Female	Negative	N/A	N/A	18.33
BTS005	33	Male	Negative	N/A	N/A	16.90
AG006	41	Male	Negative	N/A	N/A	21.48
SS007	39	Female	Negative	N/A	N/A	20.87
RKM 008	40	Male	Negative	N/A	N/A	20.68

HIV negative specimens are healthy donors

N/A = Not applicable

A

Liver Fibrosis	
Groups	Incidence (%)
Mock	0/4 (0)
cART	1/5 (20)
HIV-1	0/5 (0)
HIV-1+cART	4/6 (66.7)

B

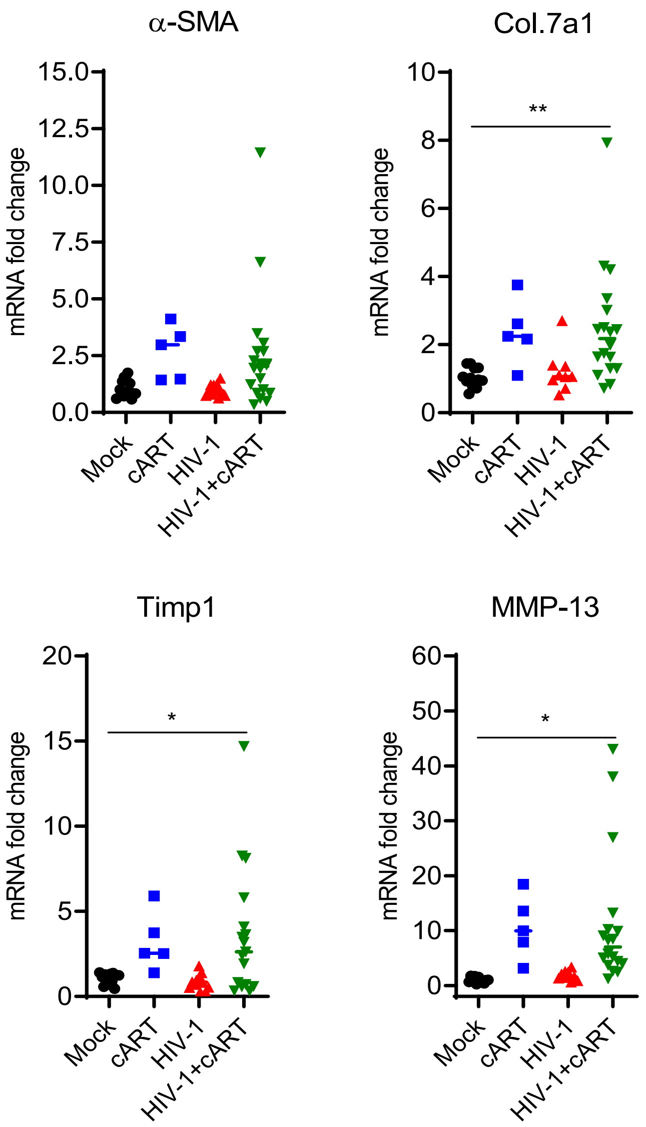

A

HIV-1+cART

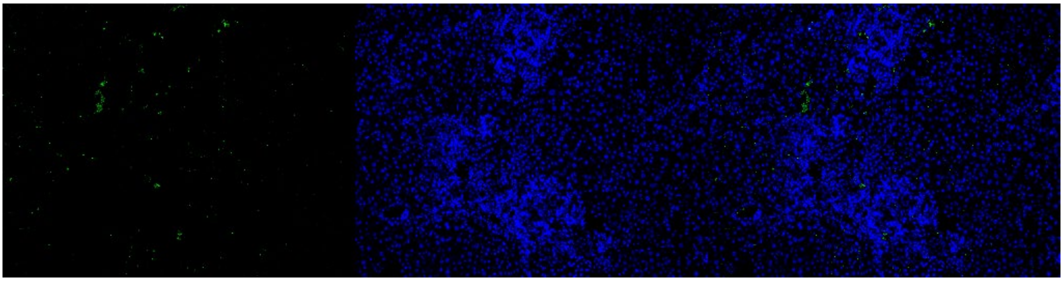

Isotype vs anti-MerTK   Nuclei   Merge

Figure S3

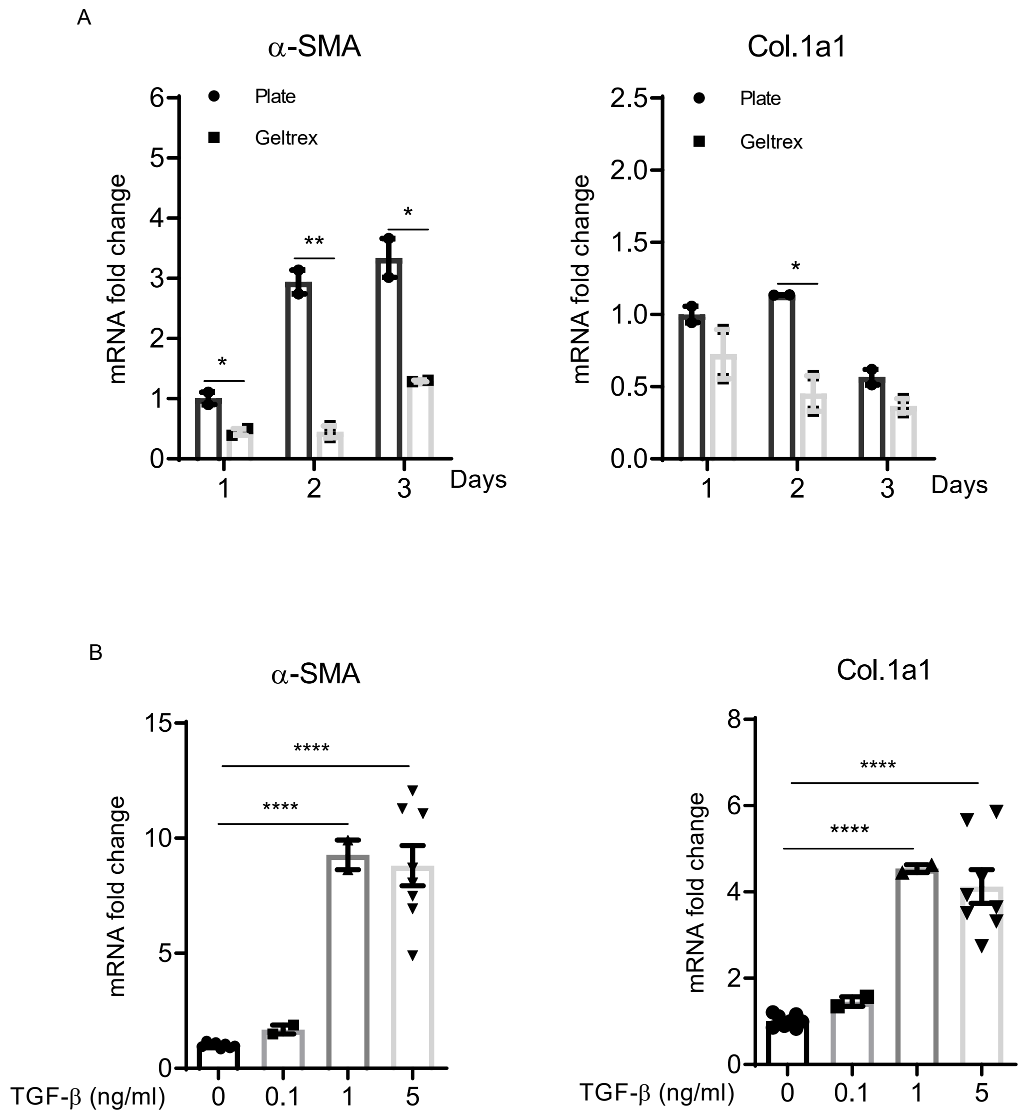

A
α-SMA
Plate
Geltrex
mRNA fold change
6
5
4
3
2
1
0
*
**
*
1
2
3
Days
Col.1a1
Plate
Geltrex
mRNA fold change
2.5
2.0
1.5
1.0
0.5
0.0
*
1
2
3
Days
B
α-SMA
mRNA fold change
15
10
5
0
****
****
TGF-β (ng/ml)
0
0.1
1
5
Col.1a1
mRNA fold change
8
6
4
2
0
****
****
TGF-β (ng/ml)
0
0.1
1
5

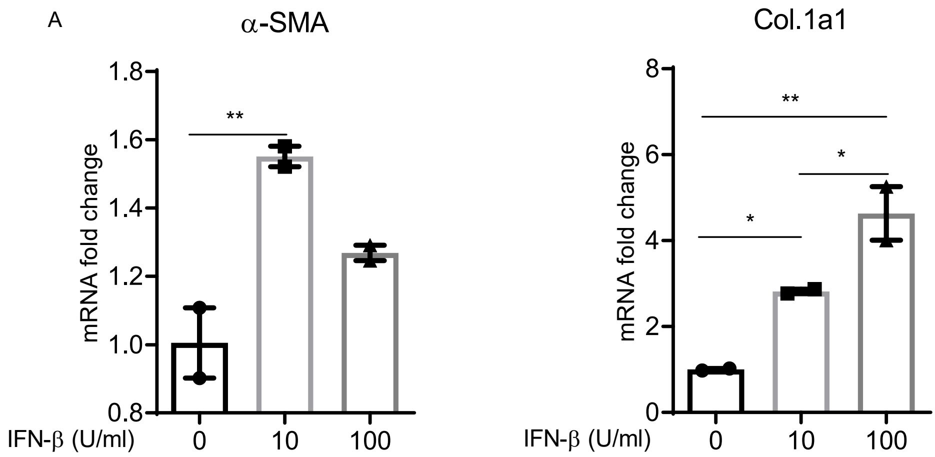
A
α-SMA
mRNA fold change
1.8
1.6
1.4
1.2
1.0
0.8
**
IFN-β (U/ml)
0
10
100
Col.1a1
mRNA fold change
8
6
4
2
0
**
*
*
IFN-β (U/ml)
0
10
100

Figure S5

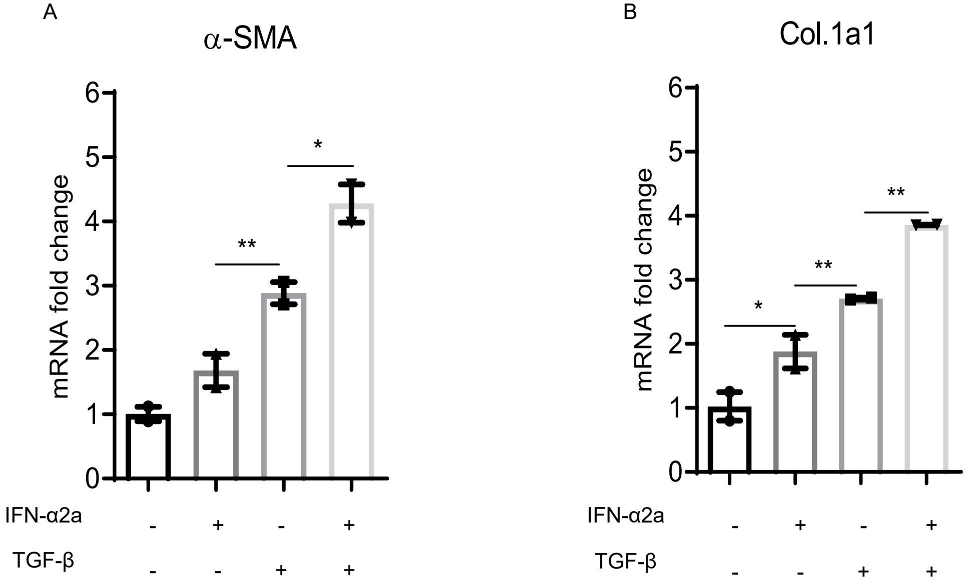
A
α-SMA
mRNA fold change
0
1
2
3
4
5
6
*
**
IFN-α2a
TGF-β
- + - +
- - + +
B
Col.1a1
mRNA fold change
0
1
2
3
4
5
6
*
**
**
IFN-α2a
TGF-β
- + - +
- - + +

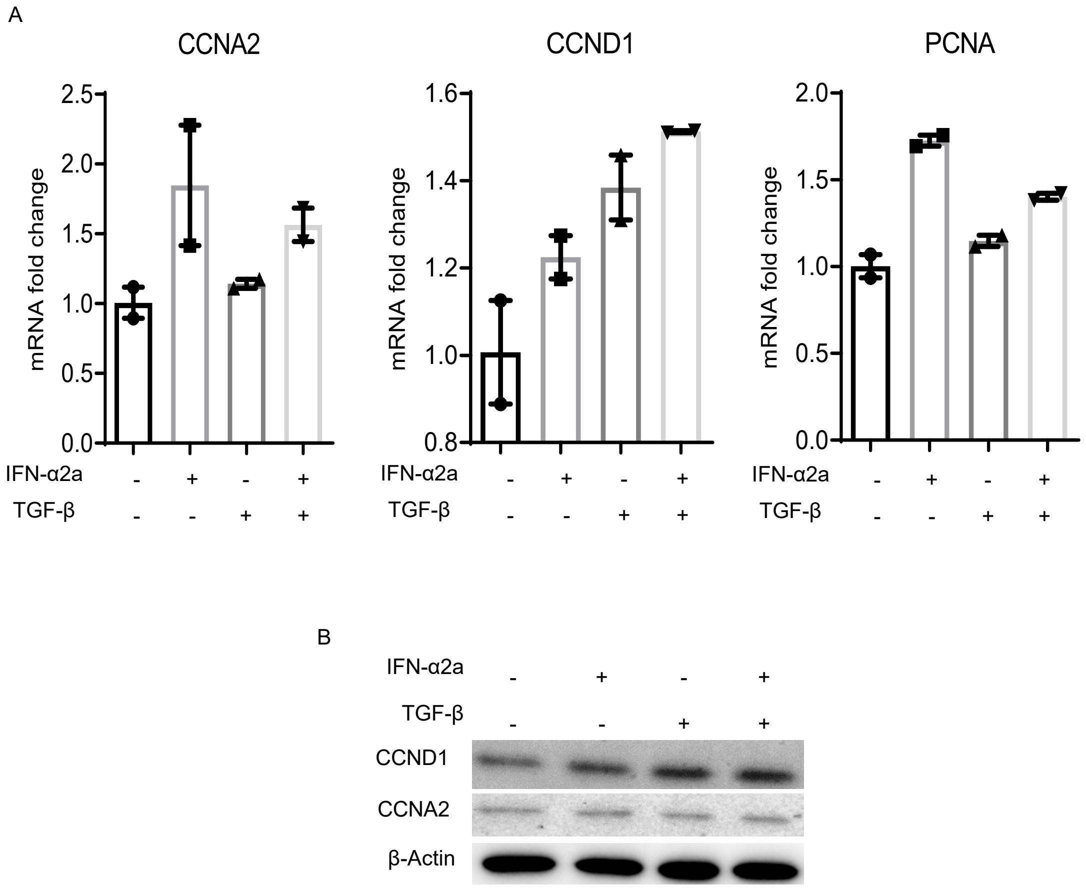
A
CCNA2
CCND1
PCNA
mRNA fold change
2.5
2.0
1.5
1.0
0.5
0.0
1.6
1.4
1.2
1.0
0.8
IFN-α2a - + - +
TGF-β - - + +
B
IFN-α2a - + - +
TGF-β - - + +
CCND1
CCNA2
β-Actin

A

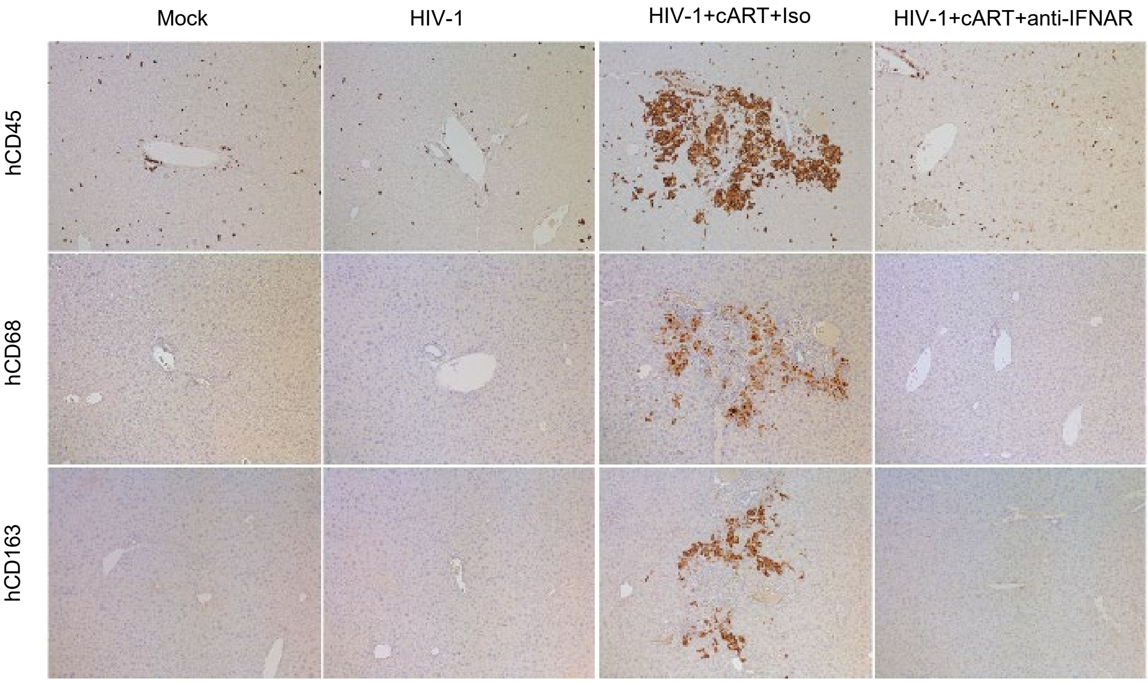
Mock
HIV-1
HIV-1+cART+Iso
HIV-1+cART+anti-IFNAR
hCD45
hCD68
hCD163

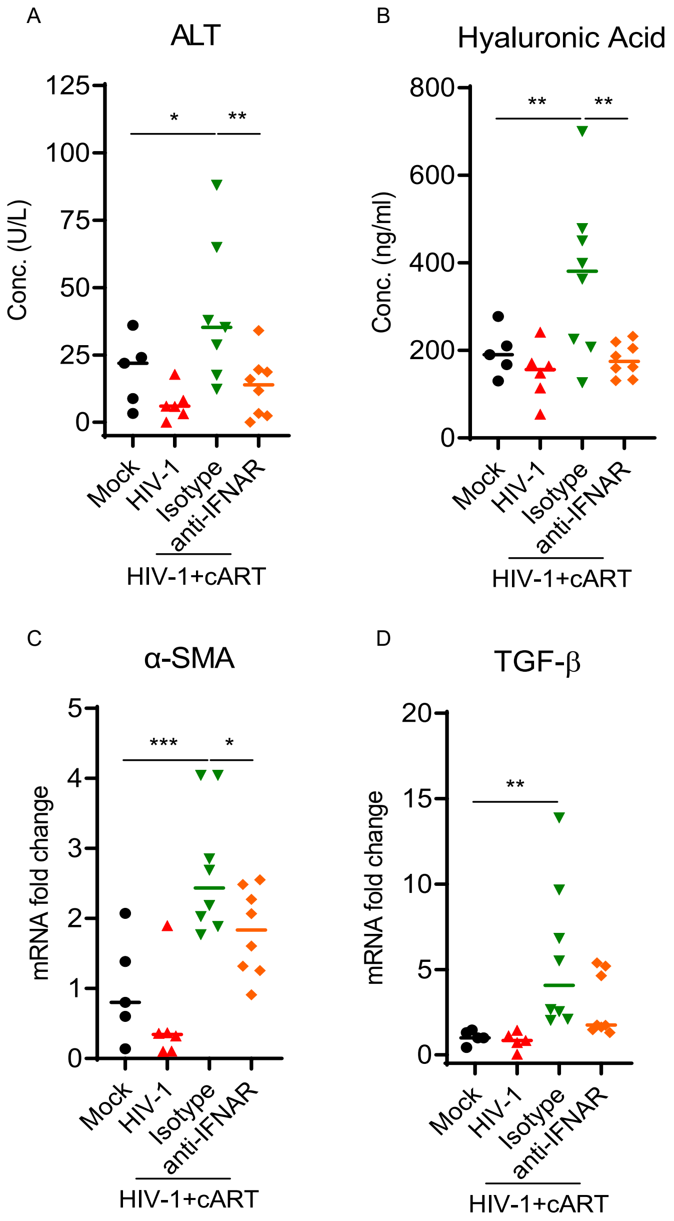
A
ALT
Conc. (U/L)
125
100
75
50
25
0
*
**
Mock
HIV-1
Isotype
anti-IFNAR
HIV-1+cART
B
Hyaluronic Acid
Conc. (ng/ml)
800
600
400
200
0
**
**
Mock
HIV-1
Isotype
anti-IFNAR
HIV-1+cART
C
α-SMA
mRNA fold change
5
4
3
2
1
0
***
*
Mock
HIV-1
Isotype
anti-IFNAR
HIV-1+cART
D
TGF-β
mRNA fold change
20
15
10
5
0
**
Mock
HIV-1
Isotype
anti-IFNAR
HIV-1+cART
